# Copy Number Variations Analysis Identifies QPRT as a Candidate Gene Associated With Susceptibility for Solitary Functioning Kidney

**DOI:** 10.3389/fgene.2021.575830

**Published:** 2021-05-17

**Authors:** Xiao Y. Zhou, Hao Y. Zheng, Li Han, Yan Wang, Li Zhang, Xiao M. Shu, Mu L. Zhang, Guan N. Liu, Lian S. Ding

**Affiliations:** ^1^Department of Obstetrics, The Affiliated Huai’an No. 1 People’s Hospital of Nanjing Medical University, Huai’an, China; ^2^College of Biological and Pharmaceutical Engineering, Nanjing Tech University, Nanjing, China; ^3^Department of Neurosurgery, The Affiliated Huai’an No. 1 People’s Hospital of Nanjing Medical University, Huai’an, China

**Keywords:** solitary kidney, DNA copy number variations, pentosyltransferases, urogenital abnormalities, cell cycle

## Abstract

**Background:**

The lack of understanding of molecular pathologies of the solitary functioning kidney makes improving and strengthening the continuity of care between pediatric and adult nephrological patients difficult. Copy number variations (CNVs) account for a molecular cause of solitary functioning kidney, but characterization of the pathogenic genes remains challenging.

**Methods:**

In our prospective cohort study, 99 fetuses clinically diagnosed with a solitary functioning kidney were enrolled and evaluated using chromosomal microarray analysis (CMA). The genetic drivers for the pathogenic CNVs were analyzed. We characterized QPRT localization in fetal kidneys using immunohistochemistry and its expression in adult kidneys using quantitative RT-PCR. Further, QPRT was knocked down using siRNA in human embryonic kidney (HEK293T) cells, and the cell cycle and proliferation were tested.

**Results:**

Besides one Triple X syndrome and one Down syndrome, we identified a total of 45 CNVs out of 34 subjects. Among the 14 pathogenic CNVs, CNV 16p11.2 reached the highest number of records with the phenotype of kidney anomalies in the Decipher database. Among the 26 genes within the 16p11.2 region, as a key enzyme for nicotinamide adenine dinucleotide (NAD+) biosynthesis, QPRT was distinctly localized in renal tubules but was barely observed in renal interstitial and glomeruli in fetal kidneys. The loss of QPRT prevented cells’ efficient transition into S phase, affected cell-cycle progression, and abrogated proliferation of human embryonic kidney cells.

**Conclusion:**

Our data suggest that QPRT is a candidate gene associated with susceptibility for solitary functioning kidney. The CNVs discovered in our study exhibit great potential for future applications in genetic counseling and pregnancy management.

## Introduction

Congenital anomalies of the kidney and urinary tract (CAKUT), defined as a wide range of morphogenic malformations of the kidney and the urinary tract, are the predominant causes of pediatric end-stage renal diseases (ESRD). CAKUT are present at birth with a ratio of 3–7 out of 1000 newborns, and effecting 40%–50% of pediatric and 7% of adult ESRD (Nicolaou et al., 2015). Within the vast spectrum of CAKUT, solitary functioning kidney is one of the distinct conditions. The development of kidney and urinary tract requires a coordinately temporal and spatial interaction between the metanephric mesenchyme (MM) and the ureteric bud (UB) (Little et al., 2016). Any failure of interaction between the MM and the UB might lead to a solitary functioning kidney. According to macroscopic and microscopic anatomic features, solitary functioning kidney results from the following malformations of renal developments: impairment of the renal parenchyma, abnormalities of the embryonic migration of the kidneys, and absence of the kidney (Vieira et al., 2018). Phenotypes underlying a congenital solitary functioning kidney include absence or underdevelopment of a kidney, such as unilateral renal agenesis/aplasia (URA) (Woolf and Hillman, 2007), renal hypodysplasia (RHD), and multicystic dysplastic kidney (MCDK). In addition, a solitary functioning kidney can also be acquired after nephrectomy because of recurrent infections or severe hydronephrosis, like vesicoureteral reflux (VUR), pelviureteric junction obstruction (PUJO), megaureter, and duplex kidney (Westland et al., 2014). Diagnosis of solitary functioning kidney requires detailed physical examination, intravenous pyelography, ultrasonography, magnetic resonance imaging (MRI), or computed tomography (CT) examination, as well as the aid of the new technologies such as next-generation sequencing and chromosome microarrays. A better understanding of the pathogenesis of solitary functioning kidney can assist physicians in improving prenatal diagnosis, genetic counseling, and clinical management.

Identification of rare CNVs and new candidate genes can shed light on the mechanisms of solitary functioning kidney. However, due to CNVs usually affecting numerous dosage-sensitive genes (Rice and McLysaght, 2017), the identification of the culprit genes remains challenging. To date, more than 75 genes contribute to RHD, which contribute to 10%–15% of cases (Sanna-Cherchi et al., 2017). As one of the highest allele frequencies of pathogenic CNVs, 16p11.2 CNV has shown an association with the deficits in genitourinary (Haller et al., 2018), neurocognitive (Weiss et al., 2008; McCarthy et al., 2009; D’Angelo et al., 2016; LeBlanc and Nelson, 2016), cardiac (Ghebranious et al., 2007; Zhu et al., 2016), and metabolic (Bochukova et al., 2010; Walters et al., 2010; Jacquemont et al., 2011; Arbogast et al., 2016) development. Commonly 16p11.2 CNV harbors around 600 kb region that consists of 26 genes. Among these genes, MYC-Associated Zinc finger protein (MAZ) is a haploinsufficient transcription factor for genitourinary development (Haller et al., 2018). The Maz knockout mouse models have shown that the survival rates were dependent on Maz copy number, and homozygous loss resulted in high penetrance of CAKUTs. The loss of TBX6, encoding T-Box transcription factor, contributes substantively to the complex traits of sporadic congenital scoliosis (Wu et al., 2015).

Notably, QPRT within 16p11.2 locus shows the highest expression in kidney (Fagerberg et al., 2014). Nevertheless, the contributions that QPRT makes to kidney pathogenesis remains elusive. It has been reported that mutations of genes involved in *de novo* NAD^+^ synthesis (e.g., QPRT) cause multiple malformations (Shi et al., 2017). QPRT reduction contributes to higher acute kidney injury (AKI) susceptibility through depressing renal NAD^+^ and increasing urinary quinolinate, implying its role as a mediator of renal stress resistance (Poyan Mehr et al., 2018). Further, the QPRT knockout mice confirm the elevation of quinolinic acid level both in the brain (Fukuoka et al., 2012) and urine (Terakata et al., 2012). When the *qprt* knockout mice are fed an NiA-free diet, the mice show niacin deficiency. Compared with the group fed a complete diet, the body weight of the NiA-free diet group, especially the kidney weight, was significantly lower (Terakata et al., 2012). In addition, QPRT heterozygous mice show increased susceptibility to renal ischemia reperfusion injury than wild-types (Ralto et al., 2020). Therefore, it is reasonable to hypothesize that QPRT is a candidate gene associated with susceptibility for the kidney disease. However, augmentation of NAD^+^ using exogenous nicotinamide may protect the kidney against diverse stressors. In addition, QPRT inhibits spontaneous cell death by suppressing overproduction of active-caspase-3 (Ishidoh et al., 2010). QPRT regulates the genes and gene networks related to neuronal differentiation of SH-SY5Y cells *in vitro*, suggesting the function of QPRT in the pathogenesis of autism spectrum disorders in Chr16p11.2 deletion carriers (Haslinger et al., 2018).

In this study, we evaluated the role of rare CNVs by using microarrays in 99 patients enrolled with a solitary functioning kidney. This study establishes an analytical pipeline to define the CNVs and provides novel candidate genes for solitary functioning kidney for the follow-up validation. Here, we hypothesized that QPRT is implicated as a candidate gene associated with susceptibility for solitary functioning kidney and that QPRT loss results in alterations of cell proliferation and death. To test this hypothesis, QPRT was knocked down using siRNA in HEK293T cells, and functional assays were performed to investigate the potential mechanism of QPRT involvement in cell cycle and proliferation. These findings imply QPRT as a potential therapeutic target in solitary functioning kidney, and have implications for therapeutic approaches such as niacin treatment.

## Materials and Methods

### Participants

The cohort of 99 pregnant Chinese women with their fetuses diagnosed with a solitary functioning kidney was enrolled from 2008 until 2017. Informed consent was obtained from all participants, and institutional approval (No. 2019190) was given by the medical ethical committee of the affiliated Huai’an No. 1 People’s Hospital of Nanjing Medical University, China. All pregnancies with a diagnosis of solitary functioning kidney by using ultrasound examinations were included. Those patients who did not have solitary functioning kidney were excluded from the study. The ultrasound phenotypes of a solitary functioning kidney include (1) RHD: the shadow of one side of the kidney significantly shrunken, with a smaller renal pelvis and calyxes instead of normal shapes; compensatory hypertrophy of the contralateral kidney; the normal position of both sides of the ureteric orifices. (2) Unilateral RA: non-visualization of one kidney with normal bladder and amniotic fluid; single renal artery; compensatory hypertrophy of the contralateral kidney. Bilateral RA: non-visualization of both kidneys and bladder in combination with anhydramnios; failure to visualize renal arteries. (3) MCDK: the presence of interfaces between cysts; non-medial location of the largest cyst; absence of an identifiable renal sinus or parenchymal tissue; multiplicity of oval or round cysts that do not communicate. If a prenatal ultrasound scan revealed that the fetus had solitary functioning kidney, fetal samples were obtained using amniocentesis or tissue or cord blood sampling.

### Chromosomal Microarray Analysis

Genomic DNA was extracted from samples following standard protocols of QIAamp DNA Mini Kit (Qiagen, Germany). Human cyto12 SNP array (Illumina, San Diego, CA, United States) covering approximately 300,000 SNP probes was applied for the whole-genome scan. Molecular karyotype analysis was performed using KaryoStudio V 1.4.3.0 (Illumina). Results were interpreted using the UCSC^1^ GRCh37/hg19 assembly and were compared with data recorded in the Database of Genomic Variants (DGV^2^), DatabasE of genomiC varIation and Phenotype in Humans using Ensembl Resources (DECIPHER^3^), and Online Mendelian Inheritance in Man (OMIM^4^). According to the American College of Medical Genetics (ACMG) standards and guidelines for interpretation of copy number variants, chromosomal microdeletions/microduplications were classified into three categories: pathogenic CNVs, variants of uncertain significance (VOUS), and benign CNVs. CNVs were defined as pathogenic if (1) the CNVs were documented as clinically significant in multiple peer-reviewed publications, regardless of its penetrance and expressivity, or (2) the CNVs overlapped with well-established clinical significance. CNVs that have been reported as benign variants in multiple peer-reviewed publications or databases were considered benign. CNVs that did not fit any of the above criteria were considered as VOUS. Fetuses with pathogenic CNVs were suggested for termination of pregnancy, while the remaining fetuses were required for follow-up until birth.

### Immunohistochemistry (IHC)

Immunohistochemistry was performed on slides containing paraffin sections. Slides were deparaffinized by xylene followed by serial rehydration in ethanol. Antigen retrieval was performed by heating slides to 95°C for 10 min in 0.1 mol/L citric acid repair solution (pH 6). Endogenous peroxidase activity was blocked by incubating slides in 3% hydrogen peroxide for 10 min. After rinsing, the slides were blocked in 5% BSA solution and incubated in a wet box at room temperature for 30 min. The slides were incubated in primary antibody (anti-QPRT: 25174-1-AP; Proteintech Group) overnight at 4°C. Next, the biotinylated secondary antibody [Goat Anti-Rabbit IgG H&L (Biotin): ab6720; Abcam] was added to the slides for 20 min at room temperature. DAB peroxidase Substrate Kit was used for visualization, and hematoxylin was used as a counterstain. Slides were mounted following serial dehydration.

### Quantitative RT-PCR

Quantitative RT-PCR experiments were performed to estimate QPRT copy number on control adult and fetal human tissues. Each sample was assayed in triplicate for each gene by using SYBR Green PCR Master Mix on a BioRad CFX96 qPCR System. Two primer couples were employed to amplify QPRT and reference gene 36B4 (Table 1). The copy number of QPRT was calculated by dividing the yield of the QPRT gene by that of the reference gene. The reaction mix for each sample was prepared in a 96-well plate, and the plate was placed in the Biorad CFX Connect^TM^ Real-Time PCR machine. Samples were run as recommendations. Samples were pre-denatured at 95°C for 10 min, followed by 40 cycles of 95°C for 10 s, 58°C for 30 s, and 72°C for 30 s. At the end of the thermal profile, the temperature was increased slowly from 60° to 95°C to generate a dissociation curve.

**TABLE 1:** Primer sequences.

Primer’s name	Primers (F: Forward; R: Reverse)	Length (bp)
QPRT-siRNA	F: CCAUAUUUACCCAACUCAATT	
	R: UUGAGUUGGGUAAAUAUGGTT	
NC-siRNA	F: UUCUCCGAACGUGUCACGUdTdT	
	R: ACGUGACACGUUCGGAGAAdTdT	
QPRT	F: GTGGCCCTCAACACGCTG	194
	R: AGCCCTCCCAGGTCGTAGC	
36B4	F: CAGCAAGTGGGAAGGTGTAATCC	75
	R: CCCATTCTATCATCAACGGGTACAA	
QPRT validation	F: CTGAAGGTGGAAGTGGAATG	275
	R: GCAAACAGCTTGAGGGAG	
GAPDH	F: CAATGACCCCTTCATTGACC	106
	R: GAGAAGCTTCCCGTTCTCAG	

### QPRT siRNA Knockdown and Validation

We used chopchop to design the target siRNA, and then delivered for synthesis. HEK293T were plated in 6-well plates at 50,000 cells per well, and transfected using lipofectamine RNAi-MAX reagent with 10 nM final concentration of siRNA following the manufacturer’s instructions. After transfection, knockdown was validated by qRT-PCR. RNA was extracted and cDNA was synthesized according to the reverse transcription kit. The cDNA was used as a template and GAPDH was used as an internal reference. After the reaction was completed, the relative quantitative analysis of Cq value was performed by the 2^–△^^△^ Ct method to calculate the relative expression of QPRT in each group of cells. Primer sequences (QPRT validation/GAPDH) were presented in Table 1.

### Western Blotting

The QPRT knockdown (QPRT siRNA), negative control (QPRT siRNA NC), and wildtype (control) HEK293T cells were seeded in 6-well plates. Cells were lysed in lysis buffer for 30 min on ice, and then insoluble materials were removed after 15 min centrifugation at 10,000 rpm/min. Protein concentrations were determined by BCA assay (Pierce, Rockford, United States). Lysate supernatant was separated using 12% sodium dodecyl sulfate-polyacrylamide gel electrophoresis and transferred onto a PVDF membrane (IPVH00010, Millipore). The membrane was incubated with Rabbit monoclonal antibody against QPRT (ab171944; Abcam) or Mouse Anti-GAPDH antibody (1/2000; TA-08; ZSGB-BIO) followed by incubation with peroxidase-conjugated goat anti-rabbit IgG (H + L) (1/2000; ZB-2301; ZSGB-BIO) and peroxidase-conjugated goat anti-mouse IgG (H + L) (1/2000; ZB-2305; ZSGB-BIO) secondary antibody, respectively. Western blots were developed using ECL solution. Grayscale results were analyzed by “Quantity one” software.

### Cell-Cycle Assay

After trypsin digestion, HEK293T cells were incubated in 1 ml DNA Staining solution and 10 ul Permeabilization solution mixture at room temperature for 30 min in the dark. Then flow cytometry was used for cell-cycle analysis. For apoptosis study, cells were resuspended in 300 ul of pre-chilled 1× binding buffer, and treated with 3 ul of Annexin V-FITC and 5 ul of propidium iodide (PI) at room temperature for 10 min in the dark. After mixing with 200 ul of pre-chilled 1× binding buffer, cells were analyzed by flow cytometry (Ex = 488 nm; Em = 530 nm) using FITC signal detector (usually FL1) and the phycoerythrin emission signal detector (usually FL2). Annexin V-FITC indicates loss of membrane asymmetry/phosphatidylserine exposure and PI denotes nuclear condensation. When cells are double stained with Annexin V-FITC and PI, three different cell populations may be observed: live cells that do not stain with either Annexin V-FITC or PI, necrotic cells that stain with both reagents, and apoptotic cells that stain with Annexin V-FITC only. After treatment with both dyes, apoptotic cells show green fluorescence, dead cells show red and green fluorescence, and live cells show little or no fluorescence.

### Statistical Analysis

The GraphPad Prism (version 8.0.1) was used for statistical analysis. One-way ANOVA was chosen from the list of column analyses. The difference in the mean between the groups was analyzed by the *t*-test. *p* < 0.05 was considered for significant differences.

## Results

### Patient Characteristics

During 2008 to 2017, a total of 99 pregnant Chinese women with their fetuses diagnosed of a solitary functioning kidney by ultrasound were recruited in this study. Among them, seven fetuses were diagnosed as acquired solitary functioning kidney, and the remaining fetuses were matched with congenital solitary functioning kidney. As shown in Table 2, the mean gestational age of these women recruited at the time of CMA test was approximately 25.1 weeks. Accordingly, two fetuses (2.02%) were diagnosed with aneuploidy (one case with trisomy 21 and one with Triple X syndrome), and 34 fetuses (36.36%) were diagnosed with CNV (Supplementary Table 1). The mean maternal age for pregnant women with aneuploidy fetuses was 26.5 years old while that for pregnant women with CNV fetus was 28.5 years old. Moreover, the gestational age of pregnant women with CNV fetus is comparably longer than that of pregnant women with aneuploidy fetuses. The mean gestational age of these women recruited for CNV analysis was approximately 25.1 weeks. And the sex ratio among the CNV fetuses was 19:15 (female:male).

**TABLE 2:** CMA results for fetuses with a solitary functioning kidney in our prospective study.

	Number	Maternal age (years, mean)	Gestational age (weeks, mean)
Total	99	28.7	25.1
Aneuploidy	2(47,XX + 21; 47,XXX)	26.5	24.5
Copy number variation	34	28.5	28.4

*CMA, chromosomal microarray analysis.*

### Chromosomal Microarray Analysis (CMA) Analyses

**FIGURE 1:**
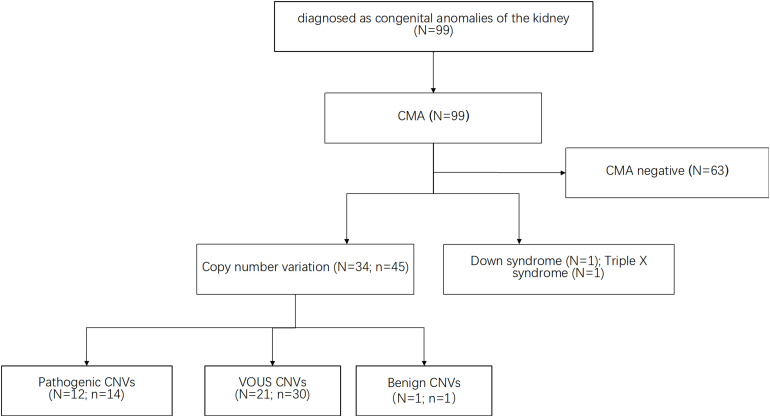
The CMA approach identifies CNVs upon subjects with a solitary functioning kidney. Several publicly available databases were used as reference resources (see the section “Materials and Methods”). CMA test upon 99 subjects identified a total of 45 CNVs out of 34 subjects. CNVs from 12 fetuses, which accounts for 11.1% of the cohort, were interpreted as pathogenic CNVs. Besides, CMA results included two cases of aneuploidy (one case of triple X cases and one case of Down syndrome). *N* indicates the number of the fetuses. *n* indicates the number of the CNVs.

All samples collected from the fetuses were analyzed by CMA. As shown in Figure 1, CMA testing upon 99 subjects identified a total of 45 CNVs out of 34 subjects (defined as CNV size > 66 kb; details are listed in Supplementary Table 1). The majority of these large CNVs were duplications (*n* = 38, 80.85%) while the rest were deletions (*n* = 7, 15.6%). According to UCSC GRCh37/hg19, these fetuses carried eight genomic imbalances with significant overlap with known genomic disorders, accounting for 8.1% of the cohort. Median large CNV size was 318,347 bp (range 66,842–5,299,999 bp). The most frequently identified CNVs were Xp22.33 duplication (*N* = 4), 2q13 duplication (*N* = 2) and 5q35.3 duplication (*N* = 2). By comparing with the data recorded in the Database of Genomic Variants (DGV), only one CNV was interpreted as benign CNV. Moreover, CNVs from 12 fetuses, which accounted for 11.1% of the cohort, matched with the records in the Database of Decipher or relevant published works for known genomic disorders or overlapped with pathogenic CNVs with a similar phenotype, which were therefore interpreted as pathogenic CNVs.

### The Architecture of Pathogenic CNVs

Among the pathogenic CNVs (*n* = 14) from 12 fetuses, eight types of CNVs from seven fetuses were found to overlap with the records in Decipher database with the same mutation type (duplication/deletion), as listed in Table 3. The size of the identified pathogenic CNVs in this study ranged from 84 kb to 521 kb. It is noteworthy that two subjects, fetus 10 and fetus 82, were diagnosed with carrying two different pathogenic CNVs. Fetus 82 had a syndrome of ectopic kidney, with 7q36.2 duplication and 9p24.1p23 deletion. Fetus 10 had a syndrome of left multicystic renal dysplasia, with 10p12.31 duplication and 12p11.23 deletion. Meanwhile, fetus 77 carried a 4p16.1 CNV which matched with three records in the Decipher database. Similarly, fetus 16 carried an 8p23.2 duplication and fetus 47 carried a 16p11.2 deletion which respectively matched with two and four records in the Decipher database. Furthermore, fetus 45 and fetus 46 both carried 22q11.2 duplications, with the phenotype being malrotated kidneys. Among the pathogenic CNVs (*n* = 14), fetus 47 carried a 16p11.2 deletion which matched with the highest records in the Decipher database. Therefore, we chose 16p11.2 for our prioritization research.

**TABLE 3:** The pathogenic CNVs in this study are matched with the records in the Decipher database for same mutation types (duplication/deletion).

Fetus ID	CNV-CMA	Band	Chromosome coordinate	Chromosome	Start	End	Duplication/deletion	CNV size (bp)	Decipher ID	Variant	Duplication/deletion	Sex	Size	Pathogenicity/contribution	Inheritance	Phenotype(s)-renal/kidney/neph/glom	Phenotype(s) -other	CNV syndromes
P77	46, XX, arr[hg19] 4p16.1(8, 212, 173-8, 437, 267) × 1	4p16.1	Chr4: 8, 212, 173–8, 437, 267	Chr4	8, 212, 173	8, 437, 267	Deletion	225094	287892	Chr4: 71, 553–8, 732, 736	Deletion	46XX	8.66 Mb	Pathogenic	*De novo* constitutive	Renal cyst	Ventricular septal defect; intrauterine growth retardation; choroid plexus cyst	NA
P77	46, XX, arr[hg19] 4p16.1(8, 212, 173-8, 437, 267) × 1	4p16.1	Chr4: 8, 212, 173–8, 437, 267	Chr4	8, 212, 173	8, 437, 267	Deletion	225094	307768	Chr4: 71, 552–18, 839, 648	Deletion	46XY	18.77 Mb	Pathogenic full	*De novo* constitutive	Abnormality of the renal pelvis, penile hypospadias	Umbilical hernia; external ear malformation; cataract, hypertelorism, iris coloboma; abnormal eyelid morphology, depressed nasal bridge, epicanthus, micrognathia, preauricular skin tag, short philtrum, up slanted palpebral fissure; fifth finger distal phalanx clinodactyly; aplasia/hypoplasia of the corpus callosum, hypoplasia of the corpus callosum; prominent protruding coccyx	NA
P77	46, XX, arr[hg19] 4p16.1(8, 212, 173-8, 437, 267) × 1	4p16.1	Chr4: 8, 212, 173–8, 437, 267	Chr4	8, 212, 173	8, 437, 267	Deletion	225094	326600	Chr4: 72, 447–11, 175, 255	Deletion	46XX	11.10 Mb	Pathogenic full	*De novo* constitutive	Chronic kidney disease	Severe intrauterine growth retardation	NA
P82	46, XX, arr[hg19] 7q36.2 (153, 529, 677-153, 763, 852) × 3	7q36.2	Chr7: 153, 529, 677–153, 763, 852	Chr7	153, 529, 677	153, 763, 852	Duplication	234175	368657	Chr7: 152, 306, 254–159, 118, 566	Duplication	46XX	6.81 Mb	Pathogenic	*De novo* constitutive	Renal dysplasia	Ventricular septal defect; intrauterine growth retardation; spinal dysraphism	NA
P82	46, XX, arr[hg19] 9p24.1p23 (8, 959, 747-9, 058, 856) × 1	9p24.1p23	Chr9: 8, 959, 747–9, 058, 856	Chr9	8, 959, 747	9, 058, 856	Deletion	99109	253970	Chr9: 2, 146, 330–9, 663, 533	Deletion	46XY	7.52 Mb	Pathogenic	*De novo* constitutive	Abnormality of the kidney, horseshoe kidney	Intellectual disability	NA
P16	46, XY, arr 8p23.2(2, 350, 511-2, 734, 916) × 3	8p23.2	Chr8: 2, 350, 511–2, 734, 916	Chr8	2, 350, 511	2, 734, 916	Duplication	384405	253970	Chr8: 487, 644–18, 600, 102	Duplication	46XY	18.11 Mb	Pathogenic	*De novo* constitutive	Abnormality of the kidney, horseshoe kidney	Intellectual disability	NA
P16	46, XY, arr 8p23.2(2, 350, 511-2, 734, 916) × 3	8p23.2	Chr8: 2, 350, 511–2, 734, 916	Chr8	2, 350, 511	2, 734, 916	Duplication	384405	278296	Chr8: 1, 765, 533–6, 939, 296	Duplication	46XX	5.17 Mb	Pathogenic	*De novo* constitutive	Ectopic kidney	Aplasia/hypoplasia of the nipples; hypertension; hypothyroidism; abnormal facial shape, reduced number of teeth; delayed speech and language development, global developmental delay, poor motor coordination; joint laxity, spinal canal stenosis	NA
P10	46, XX, dup(10p12.31, 20.76-21.00, 242K)	10p12.31	Chr10: 20, 760, 000–21, 000, 000	Chr10	20, 760, 000	21, 000, 000	Duplication	240000	354979	Chr10: 1, 48, 325–24, 786, 291	Duplication	46XY	24.64 Mb	Pathogenic full	*De novo* constitutive	Abnormality of the ureter, renal hypoplasia	Heart murmur; long eyelashes, prominent forehead; global developmental delay	NA
P47	46, XX, arr 16p11.2(29, 634, 212-30, 154, 740) × 1	16p11.2	Chr16: 29, 634, 212–30, 154, 740	Chr16	29, 634, 212	30, 154, 740	Deletion	520528	301482	Chr16: 29, 673, 954–30, 198, 600	Deletion	46XY	524.65 kb	Pathogenic partial	Unknown	Hydronephrosis, unilateral renal agenesis	Duodenal stenosis	16p11.2 deletion syndrome (Phenotype: abnormality of the face; feeding difficulties in infancy; intellectual disability; pointed chin)
P47	46, XX, arr 16p11.2(29, 634, 212-30, 154, 740) × 1	16p11.2	Chr16: 29, 634, 212–30, 154, 740	Chr16	29, 634, 212	30, 154, 740	Deletion	520528	370503	Chr16: 29, 656, 684–30, 190, 568	Deletion	46XY	533.88 kb	Pathogenic	Unknown	Renal dysplasia, unilateral renal agenesis	Global developmental delay; tracheomalacia	16p11.2 deletion syndrome (Phenotype: abnormality of the face; feeding difficulties in infancy; intellectual disability; pointed chin)
P47	46, XX, arr 16p11.2(29, 634, 212-30, 154, 740) × 1	16p11.2	Chr16: 29, 634, 212–30, 154, 740	Chr16	29, 634, 212	30, 154, 740	Deletion	520528	331267	Chr16: 29, 652, 360–30, 190, 593	Deletion	46XY	538.23 kb	Likely pathogenic	*De novo* constitutive	Abnormality of the kidney	Abnormality of coordination, autistic behavior	16p11.2 deletion syndrome (Phenotype: abnormality of the face; feeding difficulties in infancy; intellectual disability; pointed chin)
P47	46, XX, arr 16p11.2(29, 634, 212-30, 154, 740) × 1	16p11.2	Chr16: 29, 634, 212–30, 154, 740	Chr16	29, 634, 212	30, 154, 740	Deletion	520528	283293	Chr16: 29, 622, 757–30, 177, 916	Deletion	46XY	555.16 kb	Pathogenic	*De novo* constitutive	Hydronephrosis	Failure to thrive; abnormality of the thumb, hypoplasia of the radius, lower limb hypertonia, radial club hand; hypertonia; broad-based gait, global developmental delay; decreased fetal movement	16p11.2 deletion syndrome (Phenotype: abnormality of the face; feeding difficulties in infancy; intellectual disability; pointed chin)
P45	46, XX, arr 22q11.21(18, 923, 623-19, 008, 108) × 3	22q11.21	Chr22: 18, 923, 623–19, 008, 108	Chr22	18, 923, 623	19, 008, 108	Duplication	84485	304917	Chr22: 17, 029, 055–37, 959, 706	Duplication	46XX	20.93 Mb	Pathogenic	Unknown	Axial malrotation of the kidney	Patent ductus arteriosus after birth at term; iris coloboma, posterior capsular cataract; high palate, prominent nasal bridge, short nose, small anterior fontanelle, smooth philtrum; hypoplastic toenails; polyhydramnios	22q11.2 duplication syndrome (Phenotype: intellectual or learning disability, developmental delay, slow growth leading to short stature, and hypotonia)
P46	46, XX, arr 22q11.22(22, 314, 463-22, 550, 078) × 3	22q11.22	Chr22: 22, 314, 463–22, 550, 078	Chr22	22, 314, 463	22, 550, 078	Duplication	235615	304917	Chr22: 17, 029, 055–37,959,706	Duplication	46XX	20.93 Mb	Pathogenic	Unknown	Axial malrotation of the kidney	Patent ductus arteriosus after birth at term; iris coloboma, posterior capsular cataract; high palate, prominent nasal bridge, short nose, small anterior fontanelle, smooth philtrum; hypoplastic toenails; polyhydramnios	22q11.2 duplication syndrome (Phenotype: intellectual or learning disability, developmental delay, slow growth leading to short stature, and hypotonia)

*CNV, copy number variation; NA, not available.*

### Identification of Potential Genetic Drivers for Pathogenic CNV Phenotypes

**FIGURE 2:**
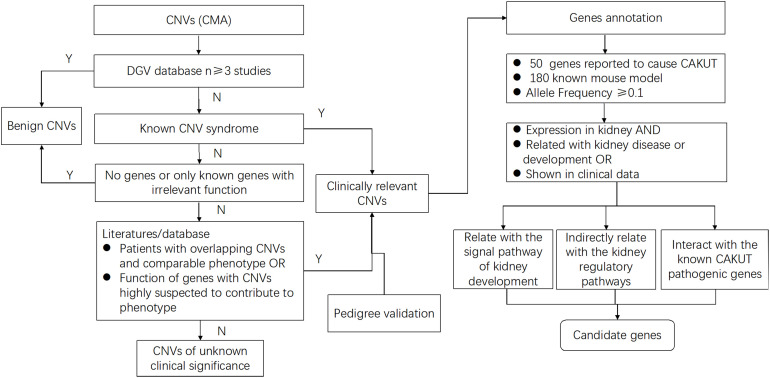
Flow diagram shows the study selection process from CNVs to candidate genes for a solitary functioning kidney. For all CNVs, deletions and duplications that showed significant overlap with pathogenic or unlikely pathogenic CNVs in public databases were included. After annotation of gene content, genes that were reported to cause solitary functioning kidneys and genes with known mouse model, as well as the genes with allele frequency ≥ 0.1, were selected. Next, the gene expression profiles in the kidney for all high-priority genes that are implicated in renal disease or other associated clinical data were evaluated. Finally, of these high-priority genes, the ones related with signal pathway of kidney development directly or indirectly, as well as the ones interacting with the known CAKUT pathogenic genes, further facilitate the selection of candidate genes. By using this systematic bioinformatic approach, we prioritized candidate genes for the solitary functioning kidney.

To define the candidate genetic drivers for the phenotype of the eight types of CNVs (Figure 2 and Table 3), a total of 37 genes were identified out of seven types of CNVs, and no genes was found in 8p23.2 locus (Table 4). To be specific, only one gene, namely PTPRD (Protein Tyrosine Phosphatase Receptor Type D), was identified in 9p24.1p23. In addition, DPP6 (Dipeptidyl Peptidase Like 6) and TOP3B (DNA Topoisomerase III Beta) were respectively identified within 7q36.2 and 22q11.22 locus. For 4p16.1 region, three genes, ACOX3, HTRA3, and SH3TC1, were identified. Moreover, we identified miRNAs (microRNAs) in the CNVs as well. For example, miR-4675 was the only gene identified in 10p12.31. Besides, two miRNAs (DGCR5 and DGCR9), as well as PRODH (Proline Dehydrogenase 1), were found to be located within the 22q11.21 region. Notably, mutation in TOP3B is associated with 22q11.2 Duplication Syndrome. DGCR5, DGCR9, and PRODH in 22q11.21 have been reported to be associated with Digeorge Syndrome. Unlike other CNVs, 16p11.2, which was selected for prioritization research, was identified to harbor 26 genes, among which seven genes were previously reported to be associated with kidney development.

**TABLE 4:** The 37 genes within these pathogenic CNVs.

CNV	Start	End	Genes
4p16.1	8,212,173	8,437,267	ACOX3
			HTRA3
			SH3TC1
7q36.2	153,529,677	153,763,852	DPP6
8p23.2	2,350,511	2,734,916	–
9p24.1p23	8,959,747	9,058,856	PTPRD
10p12.31	20,760,000	21,000,000	**MIR-4675**
16p11.2	29,634,212	30,154,740	SPN
			QPRT
			C16orf54
			ZG16
			KIF22
			MAZ
			PRRT2
			PAGR1
			MVP
			CDIPT
			SEZ6L2
			ASPHD1
			KCTD13
			TMEM219
			TAOK2
			HIRIP3
			INO80E
			DOC2A
			C16orf92
			FAM57B
			ALDOA
			PPP4C
			TBX6
			YPEL3
			GDPD3
			MAPK3
22q11.21	18,923,623	19,008,108	**DGCR5**
			**DGCR9**
			PRODH
22q11.22	22,314,463	22,550,078	TOP3B

*The bold font indicates ncRNAs. No genes in 8p23.2 locus.*

### Identification of Potential Susceptibility Genes for 16p11.2 CNV

**FIGURE 3:**
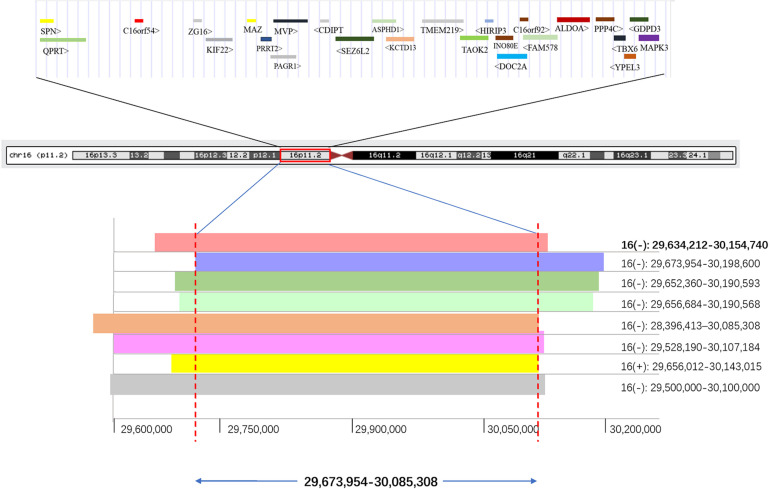
The minimal region (411 kb) of 16p11.2 CNVs in eight patients with renal anomalies from the DECIPHER database and literature was defined. QPRT occupies the minimal region of 16p11.2 CNVs in patients with solitary functioning kidneys. The first bold CNV was from this study. (+) indicates duplication; (–) indicates deletion.

Combining with a retrospective into the literature about 16p11.2 duplication/deletion syndromes, we listed the phenotype and pathogenicity of another eight kidney abnormal patients harboring 16p11.2 dosage variations besides fetus 47 in our study (Table 5). By compiling the CNVs of these nine patients into a CNV overlap map, it is easily visible that eight out of nine patients harbored a genomic space in 16p11.2 between coordinator 29,673,954 and 30,085,308 (Figure 3). The molecular mechanisms of five genes specifically involved in the diseases, MAZ, PAGR1, MVP, TBX6, and MAPK3, have been studied (Supplementary Table 2). Meanwhile, among the other three genes, only QPRT was reported to be associated with the pathogenesis of neurodegenerative disorders, which was also a syndrome of 16p11.2. And the expression profiles provided by GTEx Portal^5^ indicated that QPRT was highly expressed in kidney with an FPKM value of 37.95. Therefore, we chose QPRT as a candidate gene associated with susceptibility for solitary functioning kidney.

**TABLE 5:** Pathogenic CNVs in 16p11.2 with the phenotypes of the solitary functioning kidney in reviewed studies.

Case	CNVs	Deletion/duplication	Pathogenicity	Phenotype	References
1	Chr16: 29,634,212–301,54,740	Deletion	Pathogenic	Unilateral renal agenesis	**This study**
2	Chr16: 29,673,954–30,198,600	Deletion	Pathogenic partial	Duodenal stenosis, hydronephrosis	Decipher ID: 301482
				Unilateral renal agenesis	
3	Chr16: 29,656,684–30,190,568	Deletion	Pathogenic	Global developmental delay, renal dysplasia, tracheomalacia, unilateral renal agenesis	Decipher ID: 370503
4	Chr16: 28,733,550–28,950,951	Deletion	N/A	Patient 1: left renal agenesis, grade-IV vesicoureteral reflux, Hirschsprung disease.	Sampson et al. (2010)
5	Chr16: 28,396,413–30,085,308	Deletion	N/A	Patient 2: left renal agenesis, chronic kidney disease, chronic constipation, seizures, developmental delay.	Sampson et al. (2010)
6	Chr16: 29,528,190–30,107,184	Deletion	N/A	Congenital diaphragmatic hernia, chordae, cleft palate, polydactyly, congenital heart defect, multicystic dysplastic kidney, fusion of lower ribs, and pyloric stenosis.	Shinawi et al. (2010)
7	Chr16: 29,652,360–30,190,593	Deletion	Likely pathogenic	Abnormality of coordination, abnormality of the kidney, autistic behavior	Decipher ID: 331267
8	Chr16: 29,656,012–30,143,015	Duplication	Pathogenic	Anemia, menorrhagia, abnormality of the kidney, onychogryphosis of toe nails, autism, depression, intellectual disability, mild, obsessive–compulsive behavior	Decipher ID: 327119
9	Chr16: 29,500,000–30,100,000	Deletion	N/A	Unilateral multiple renal cysts	OMIM ID: 611913

### Expression Profile of QPRT

**FIGURE 4:**
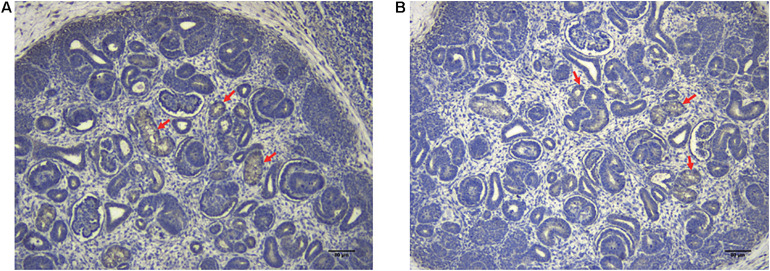
The localization of QPRT in healthy fetal renal tissues (70 days of gestation) was evaluated by immunohistochemical analyses. QPTR had strong expression in renal tubes (red arrows), whereas they were weakly or not expressed in renal interstitial and glomeruli. Scale bar: 80 μm in panels **(A,B)**.

**FIGURE 5:**
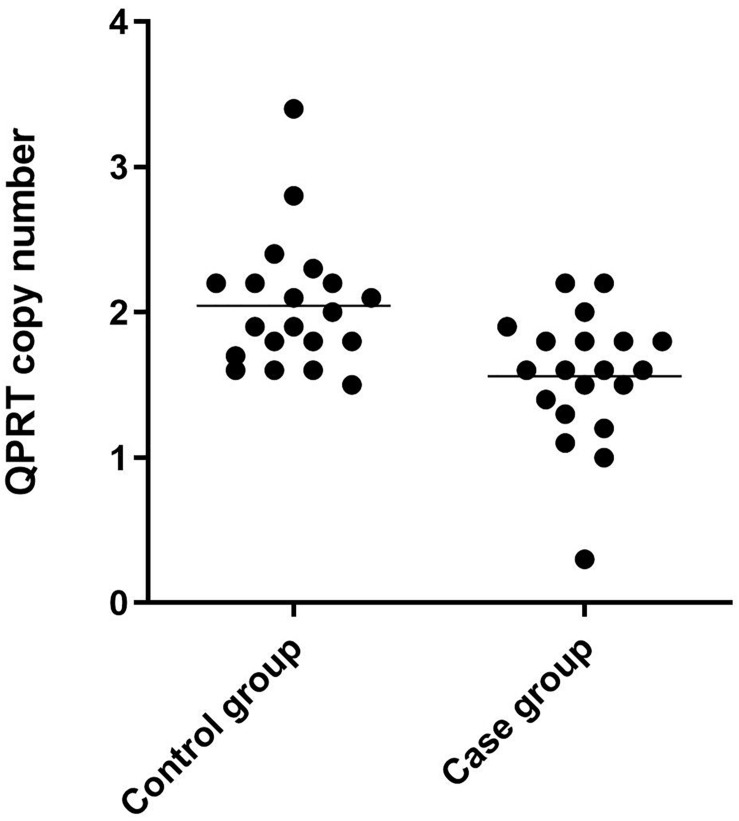
Scatter dot plot of QPRT copy number results of 40 clinical samples by qRT-PCR. The control group was composed of 20 healthy adults, and the case group consisted of the other 20 fetuses from the 99 solitary functioning kidney cohort.

Immunohistochemical staining of the healthy fetal kidney demonstrated that QPRT was distinctly detected in renal tubules while being barely observed in renal interstitial and glomeruli (Figure 4). Furthermore, we randomly selected 20 fetuses (from the cohort of solitary functioning kidney, as case group) and 20 healthy adults (as control group) for quantitative real time polymerase chain reaction (qRT-PCR) to analyze the CNV of QPRT. As shown in Figure 5, the average QPRT copy number of control group was significantly higher than that of case group. Moreover, according to the Exome Aggregation Consortium database (ExAC^6^), QPRT has a loss-of-function variant frequency of <1:1,000, and the pLI score which means the gene’s tolerance to a loss of function mutation is 0.59. Taken together, the above results implied that QPRT mutation might have an indirect role in nephrogenesis during the fetal period.

### Knockdown of QPRT in HEK293T Cells Disrupts Cell Cycle and Results in Growth Deficiency

**FIGURE 6:**
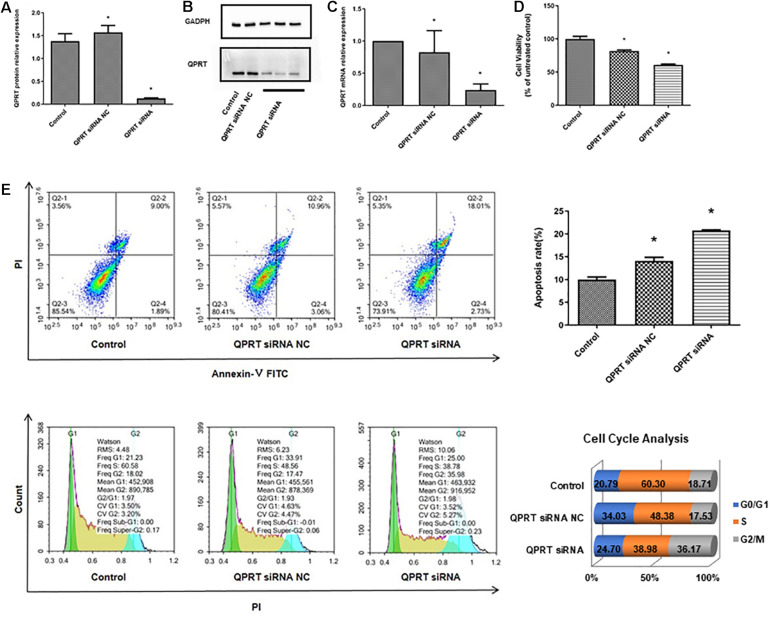
Knockdown of QPRT in HEK293 cells by using siRNA transfection inhibits growth and disrupts the process of cell cycle. The knockdown cells were validated by **(A)** quantifications of western blots, **(B)** western blot, and **(C)** qRT-PCR for QPRT. **(D)** CCK8 assays for cell proliferation after 48 h transfection were performed and showed that QPRT knockdown cells exhibited significant reduction in growth efficiency. **(E)** Apoptosis and cell cycle analysis by flow cytometry revealed that QPRT KD cells cannot enter the S phase with the same efficiency as the control cells, showing a 21% reduction in the number of cells able to enter S phase over the same time frame. NC indicates negative control group, which was transferred with empty vectors (*P-value < 0.05, vs. control group).

To verify the potential functional link between the inactivation of QPRT and renal cell development, QPRT was knocked down using siRNA in human embryonic kidney cells, and the successful knockdown of QPRT was further confirmed by western blot (Figures 6A,B) and qRT-PCR (Figure 6C). Moreover, CCK8 assay demonstrated that QPRT knockdown cells significantly exhibited reductions in proliferation (Figure 6D). Flow cytometry data revealed that the apoptosis rate of QPRT knockdown cells was significantly higher than the control groups. The QPRT knockdown cells cannot enter the S phase with the same efficiency as control cells, showing a 21% reduction in the number of cells able to enter S phase over the same time frame. Compared with the control group, the percentage of cells in the G2 phase was more significantly increased than that of the G1 phase. The reduction in the number of cells in S phase and the increased efficiency of entering G2 phase indicated that cells transfected with QPRT siRNA cannot divide properly from tetraploid to diploid (Figure 6E). Hence, cell proliferation was inhibited. Together, these findings suggest that QPRT is involved in the regulation of cell-cycle progression and proliferation of HEK cells.

## Discussion

Here our findings identify that diverse pathogenic CNVs account for 12.1% of diseases in fetuses with a solitary functioning kidney. The lack of a comprehensive understanding of the pathogenesis of solitary functioning kidney restricts the development of genetic counseling and pregnancy management. Here we proposed an *in silico* pipeline to narrow down the pathogenic CNVs to identify the possible genetic drivers for follow-up studies, and in total 14 pathogenic CNVs of 99 subjects were identified. However, the partial of familial DNA in the study was not available for CNV analysis, which limited the confirmation of genetic causation.

To define the candidate genetic drivers for the phenotype of the eight types of CNVs, a total of 37 genes were identified out of seven types of CNVs, and no genes were found in 8p23.2 locus. To be specific, only one gene, namely PTPRD (Protein Tyrosine Phosphatase Receptor Type D), was identified in 9p24.1p23. In addition, DPP6 (Dipeptidyl Peptidase Like 6) and TOP3B (DNA Topoisomerase III Beta) were respectively identified within 7q36.2 and 22q11.22 locus. For 4p16.1 region, three genes, ACOX3, HTRA3, and SH3TC1, were identified. Notably, PRODH (Proline Dehydrogenase 1) in 22q11.21 has been reported to be associated with Digeorge Syndrome, and mutation in TOP3B is associated with 22q11.2 Duplication Syndrome. As the prioritization research shows, 16p11.2 was identified to harbor 26 genes, among which seven genes were previously reported to be associated with kidney development.

Moreover, we identified ncRNAs (non-coding RNAs) in the CNVs as well. miRNA and lncRNAs are two major families of the ncRNAs, which regulate fundamental cellular processes via diverse mechanisms. The association of ncRNAs with a specific state of diseases makes them potential candidate biomarkers for the pathogenesis of human diseases. In this study, we identified miR-4675 within the locus 10p12.31 as a novel candidate susceptibility gene for kidney malformations for the very first time. To date, there was no according descriptions nor OMIM database records in respect to the functions of miR-4675. Besides, two lncRNAs (long non-coding RNA) (DGCR5 and DGCR9), which have assessed their relationship with Digeorge Syndrome and Velocardiofacial Syndrome (Gao et al., 2015), were found to be located within the 22q11.21 CNV. Intriguingly, in addition to the structural genes within each CNV, we also identified one intergenic CNV 8p23.3. We speculated this rare intergenic CNV might regulate the flank candidate genes which function in kidney development.

The fact that the average QPRT copy number of control group was significantly higher than that of case group suggests that the low copy number of QPRT is one of the attributes of the solitary functioning kidney. Since loss of QPRT leads to defective phenotypes (e.g., AKI), this study aims to reveal the contributions QPRT makes to the phenotypes of 16p11.2 deletion. However, we cannot exclude MAZ as a culprit. Here, we speculate that QPRT might act in an interactive manner to modify the culprit’s penetrance and expressivity, or that multiple genes including QPRT may act together as drivers with reciprocal interactions.

Given that QPRT can inhibit apoptosis in malignancies (Ishidoh et al., 2010; Sahm et al., 2013; Ullmark et al., 2017) and neuronal cells (Haslinger et al., 2018), we questioned whether knockdown of QPRT results in disrupted HEK293T cell growth. However, HEK293T cells are not of embryonic kidney origin but adrenal gland origin, which is a limitation of our study. In the future, to confirm these results, further studies in more types of kidney cells are needed. Consistent with the previous results, this study shows that QPRT deficiency reduces the cells proliferation, impedes cell cycle progression, prevents the cells transition into S phase effectively, abrogates cell division, and promotes cell death. Moreover, the exact localization of QPRT in fetal kidney implies the regulation of QPRT in human renal tubules development. However, Katsumi’s research didn’t show the kidney histological features of the QPRT knockout mice. Therefore, analyzing the QPRT knockout mouse model could help elucidate the molecular etiology of QPRT loss at kidney malformations.

To elucidate the underlying mechanisms of QPRT-knockdown-induced kidney cell death in our study, we investigated the expression of WNT4 and WNT11, as well as WT1. Previous studies reported that dysregulations of WNT expression cause a variety of developmental disorders, such as CAKUT (Halt and Vainio, 2014). Specifically, WNT4 is essential for nephrogenesis through regulating kidney tubule induction (Itaranta et al., 2006). Impairment of WNT11 leads to kidney tubular abnormalities and secondary glomerular cystogenesis (Nagy et al., 2016). Moreover, Wilms Tumor 1 (WT1) negatively regulates WNT/beta-catenin pathway in nephrogenesis (Kim et al., 2010). In leukemic cells, QPRT is a direct target gene of WT1, where WT1 binds to a conserved site of the first intron of the QPRT gene (Ullmark et al., 2017). As shown in Supplementary Figure 1, WNT4 was significantly increased in QPRT reduction cells. Yet, the expression of WNT11 did not show a significant decrease. Consistent with the study in leukemic cells, the dramatic down-regulation of potent developmental regulators WT1 was observed in QPRT-suppressed human kidney cells (see Supplementary Figure 1), further strengthening the negative role of WT1 in regulating WNT/beta-catenin pathway. Together, these data indicate that WNT pathway is likely to play a role in the QPRT-deleted kidney cells. Nevertheless, to comprehensively understand the mechanism of QPRT in kidney development, the cDNA of the QPRT knockdown cells needs to be subjected to expression microarrays consisting of the majority representative WNT pathway probes.

Genetic diagnosis of solitary functioning kidney facilitates physicians to correctly diagnose the extent of the disease to improve prognostic counseling and personalized disease management. By understanding the molecular pathways which are required for normal kidney development, new therapies can be developed to neutralize the genetic imbalances of solitary functioning kidney in the hopes that fewer infants will be born with these defects.

## Data Availability Statement

The datasets for this article are not publicly available due to concerns regarding participant/patient anonymity. Requests to access the datasets should be directed to the corresponding author.

## Ethics Statement

The studies involving human participants were reviewed and approved by the Medical Ethical Committee of the Affiliated Huai’an No. 1 People’s Hospital of Nanjing Medical University, China. Written informed consent to participate in this study was provided by the participants’ legal guardian/next of kin. Written informed consent was obtained from the individual(s), and minor(s)’ legal guardian/next of kin, for the publication of any potentially identifiable images or data included in this article.

## Author Contributions

XZ, HZ, LH, YW, LZ, XS, MZ, and LD recruited patients and collected clinical information. XZ and HZ performed molecular analyses. LH, YW, and LZ performed IHC experiments. XZ, HZ, LH, YW, LZ, XS, MZ, GL, and LD performed data analysis and interpretation. XZ, GL, and LD designed the study and wrote the manuscript. All authors contributed to the article and approved the submitted version.

## Conflict of Interest

The authors declare that the research was conducted in the absence of any commercial or financial relationships that could be construed as a potential conflict of interest.

## Footnotes

1 http://genome.ucsc.edu/

2 http://projects.tcag.ca/variation/

3 http://decipher.sanger.ac.uk/

4 http://www.ncbi.nlm.nih.gov/omim

5 https://gtexportal.org

6 http://exac.broadinstitute.org/

## Supplementary Material

Supplementary Figure 1 The expression profiles of WNT4, WNT11, and WT1 in the QPRT knockdown cells at mRNA level were evaluated. QPRT siRNA NC group indicates negative control group, which was transferred with empty vectors. QPRT siRNA group means QPRT was knocked down by using siRNA. ^∗∗^*P* ≤ 0.01; ^∗∗∗^*P* ≤ 0.001; ^****^*P* ≤ 0.0001.

Supplementary Table 1 Cytogenetic and clinical features of the 34 fetuses diagnosed as solitary functioning kidney in this study.

Supplementary Table 2 Summary of the 26 genes within the shared region of 16p11.2 CNVs.

## Abbreviations

QPRT: quinolinate phosphoribosyltransferase
CMA: chromosomal microarray analysis
CNV: copy number variation.
