# Supplementary material for: Copy Number Variations Analysis Identifies QPRT as a Candidate Gene Associated With Susceptibility for Solitary Functioning Kidney
Source: Front Genet. 2021 May 17;12:575830. doi: 10.3389/fgene.2021.575830 (PMC8165445; doi:10.3389/fgene.2021.575830)

Figure S1 The expression profiles of WNT4, WNT11 and WT1 in the QPRT knockdown cells at mRNA level were evaluated. QPRT siRNA NC group indicates negative control group, which was transferred with empty vectors. QPRT siRNA group means QPRT was knocked down by using siRNA. **P-value ≤ 0.01; ***P-value ≤ 0.001; ****P-value ≤ 0.0001.

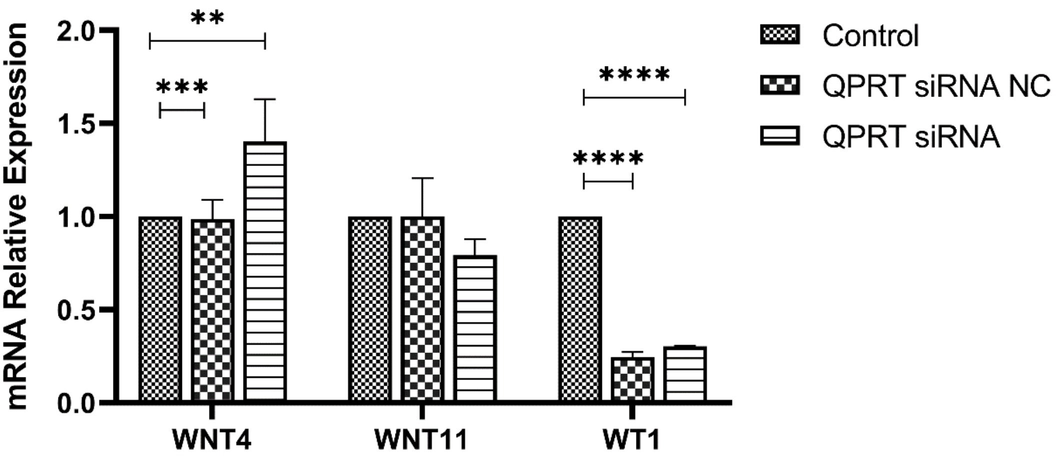

Supplement: Supplementary Figure 1 — The expression profiles of WNT4, WNT11, and WT1 in the QPRT knockdown cells at mRNA level were evaluated. QPRT siRNA NC group indicates negative control group, which was transferred with empty vectors. QPRT siRNA group means QPRT was knocked down by using siRNA. ∗∗P ≤ 0.01; ∗∗∗P ≤ 0.001; ****P ≤ 0.0001. [file Data_Sheet_1.PDF]
