# Supplementary material for: Copy Number Variations Analysis Identifies QPRT as a Candidate Gene Associated With Susceptibility for Solitary Functioning Kidney
Source: Front Genet. 2021 May 17;12:575830. doi: 10.3389/fgene.2021.575830 (PMC8165445; doi:10.3389/fgene.2021.575830)
Supplement: Supplementary Table 1 — Cytogenetic and clinical features of the 34 fetuses diagnosed as solitary functioning kidney in this study. [file Data_Sheet_2.PDF]

Table S1. Cytogenetic and clinical features of the 34 fetuses diagnosed as solitary functioning kidneys in this study.

Patient ID	Maternal Age (weeks)	Gestational Age (weeks)	Samples	CNV	CNV Type	CNV size (bp)	Clinical Manifestations	Known Genomic Disorders (All coordinates are according to hg19)
95	28	29+1	Skin tissue	1q21.2	deletion	193126	MCDK	
50	19	30+4	amniotic fluid	1p36.21	duplication	336983	MCDK (left)	1p36 duplication
83	30	25+2	amniotic fluid	2q13	duplication	482154	MCDK (left)	
				Xp21.1	duplication	737756		
84	34	22+3	amniotic fluid	16p12.3	duplication	129983	MCDK (right)	
				2q13	duplication	106461		
				5p15.33	duplication	211340		5p distal duplication
77	27	24+	amniotic fluid	4p16.1	deletion	225094	MCDK (right)	
96	27	19	Skin tissue	4q31.3	duplication	915147	RHD (left)	
				Xp22.33	duplication	188062		
88	33	23+5	amniotic fluid	6q14.1	duplication	230473	URA (right)	
				6q14.1		134777		
3	25	25+3	amniotic fluid	7q11.23	duplication	5299999	RHD (right)	7q11.23 duplication syndrome; Williams-Beuren Syndrome (WBS);
75	30	25	amniotic fluid	7q36.1	duplication	476330	RHD (right)	
80	25	24+3	amniotic fluid	7q11.21	duplication	388218	RHD (right)	
78	29	24	amniotic fluid	7p21.2	deletion	66842	MCDK (right)	
				5q35.3	duplication	163691		
16	28	23	amniotic fluid	8p23.2	duplication	384405	MCDK (right)	
72	25	21	amniotic fluid	9p23	duplication	824183	URA (right)	
10	33	24+5	amniotic fluid	10p12.31	duplication	242000	MCDK (left)	
				12p11.23	deletion	500000		
86	39	25+5	amniotic fluid	10p11.23	duplication	121234	URA	
				Xq27.2	duplication	122846		
65	27	25	amniotic fluid	13q12.11	duplication	516935	RHD (left)	
				16q23.2	duplication	431100		
22	21	27+1	amniotic fluid	15q11.2	duplication	778770	MCDK (right)	15q11.2 Prader-Willi/Angelman region reciprocal duplication
32	29	25+2	Skin tissue	15q26.3	duplication	284267	MCDK (bilateral)	15q26 overgrowth syndrome duplication
97	29	23+5	Skin tissue	15q11.1q11.2	duplication	876738	MCDK (right)	
47	33	28	amniotic fluid	16p11.2	deletion	520528	URA (left)	16p11.2 deletion
40	27	28+2	amniotic fluid	16q24.3	duplication	215531	URA (right)	
63	26	21	amniotic fluid	16q23.3q24.1	duplication	990447	URA (right)	
35	27	25	amniotic fluid	Xp22.31	duplication	363078	MCDK (right)	
				Xp22.31		497668		
34	31	24+1	Skin tissue	Xp22.33	duplication	202975	MCDK (bilateral)	
42	24	32+3	Skin tissue	Xp22.33	duplication	466670	RHD (bilateral)	
49	24	26+3	Skin tissue	Xp22.33	duplication	642041	URA (left), RHD (right)	
60	35	24+4	amniotic fluid	Xq21.31	duplication	461820	MCDK (right),	
29	29	29+3	amniotic fluid	1q43	duplication	361409	megacalyectasis	
25	36	29+5	amniotic fluid	5q35.3	duplication	180933	Hydronephrosis	
82	26	24+1	amniotic fluid	7q36.2	duplication	234175	ectopic kidney	
				9p24.1p23	deletion	99109		
62	25	22	amniotic fluid	13q14.11	duplication	318347	ectopic kidney (left)	
81	33	30+2	amniotic fluid	16q24.3	deletion	81359	ectopic kidney (right)	
45	29	26+4	amniotic fluid	22q11.21	duplication	84485	duplex kidney (left)	22q11.2 duplication syndrome;
46	27	24+2	amniotic fluid	22q11.22	duplication	235615	duplex kidney (right)	22q11.3 duplication syndrome;
