# Supplementary material for: Copy Number Variations Analysis Identifies QPRT as a Candidate Gene Associated With Susceptibility for Solitary Functioning Kidney
Source: Front Genet. 2021 May 17;12:575830. doi: 10.3389/fgene.2021.575830 (PMC8165445; doi:10.3389/fgene.2021.575830)
Supplement: Supplementary Table 2 — Summary of the 26 genes within the shared region of 16p11.2 CNVs. [file Data_Sheet_3.PDF]

Table S2 Summary of the 26 genes within the shared region of 16p11.2 CNVs.

Gene	Gene ID	the maximum tissue-specific FPKM value	FPKM value in kidney	Localization	OMIM-Gene	OMIM-Phenotype	References (related with kidney)	Molecular regulation of kidney development
SPN	6693	38.629	0.243	plasma membrane, extracellular	182160	NA	NA	NA
QPRT	23475	77.899	37.95	cytosol	606248	NA	Acute kidney injury [27, 31]	NA
C16orf54	283897	28.95	0.36	plasma membrane	NA	NA	NA	NA
ZG16	653808	252.76	0.016	extracellular, golgi apparatus	617311	NA	NA	NA
KIF22	3835	29.515	8.668	cytoskeleton, cytosol, nucleus	603213	603546	NA	NA
MAZ	4150	68.321	27.388	nucleus	600999	NA	Genitourinary development [13]<br>Renal collecting duct system [32]	Wnt signaling pathway [33]
PRRT2	112476	3.563	3.563	plasma membrane	614386	602,066,128,200,605,000	NA	NA
PAGR1	79447	11.526	4.694	nucleus,cytosol	603501	NA	Renal Cell Carcinoma [34]	RhoA-ROCK Signaling [34]

MVP	9961	110.36	71.643	cytoskeleton,nuleus, cytosol, extracellular	605088	NA	Nephroblastomas, Renal cell carcinoma [35-37]	regulating the MAP kinase, JAK/STAT and phosphoinositide 3-kinase/Akt signaling pathways [38]
CDIPT	10423	77.306	40.423	plasma membrane, endoplasmic reticulum, golgi apparatus	605893	NA	NA	NA
SEZ6L2	26470	84.837	2.876	plasma membrane, extracellular, endoplasmic reticulum	616667	NA	NA	NA
ASPHD1	253982	59.476	1.589	nucleus, cytosol	NA	NA	NA	NA
KCTD13	253980	32.407	4.074	KCTD13, cytosol	608947	NA	NA	NA
TMEM219	124446	80.283	41.538	plasma membrane, extracellular	NA	NA	NA	NA
TAOK2	613199	24.488	11.675	cytoskeleton, nucleus, cytosol, plasma membrane	NA	NA	NA	NA

HIRIP3	603365	44.911	6.869	nucleus, cytosol	NA	NA	NA	NA
INO80E	283899	40.107	16.947	nucleus, cytosol	NA	NA	NA	NA
DOC2A	604567	20.934	0.804	nucleus, cytosol, lysosome	NA	NA	NA	NA
C16orf92	146378	11.471	0	plasma membrane, extracellular, cytosol	NA	NA	NA	NA
FAM57B	615175	25.348	0.012	endoplasmic reticulum, plasma membrane, golgi apparatus	NA	NA	NA	NA
ALDOA	226	1352.67	205.84	cytosol, extracellular, cytoskeleton	103850	611881	Renal cell carcinoma [39,40]<br>Kidney injury [41],<br>Hypernephroid carcinoma [42]	Wnt/β-catenin signaling pathway [39,43]
PPP4C	83723	75.522	32.953	cytoskeleton, nucleus, cytosol, plasma membrane	615175	NA	NA	NA
TBX6	6911	4.418	0.874	nucleus	602427	122600	Renal agenesis [44,45], CAKUT [46]	Notch signaling pathway [44]
YPEL3	83719	127.556	23.065	nucleus, extracellular	609724	NA	NA	NA

GDPD3	79153	33.944	6.92	endoplasmic reticulum, nucleus, cytosol	616318	NA	NA	NA
MAPK3	5595	67.47	18.163	plasma membrane, cytoskeleton, mitochondrion, nucleus, endosome, cytosol, golgi apparatus, extracellular	601795	NA	End-stage renal disease [47], Unilateral ureteral obstruction [48]	MAPK/ERK Signaling

Abbreviations: NA, not available. FPKM, Fragments Per Kilobase per Million, a normalized estimation of gene expression based on RNA-seq data; OMIM, Online Mendelian Inheritance in Man, a comprehensive compendium of human genes and genetic phenotypes that is freely available and updated daily.
